# The Avon Longitudinal Study of Parents and Children - A resource for COVID-19 research: Home-based antibody testing results, October 2020. An emphasis on self-screening at a population level

**DOI:** 10.12688/wellcomeopenres.16616.2

**Published:** 2021-12-02

**Authors:** Kate Northstone, Daniel Smith, Claire Bowring, Amanda Hill, Richard Hobbs, Nicholas Wells, Nicholas J. Timpson

**Affiliations:** 1 ALSPAC, Department of population Health Sciences, Bristol Medical School, Unviersity of Bristol, Bristol, BS8 2BN, UK; 2 MRC Integrative Epidemiology Unit, Department of Population Health Sciences, Bristol Medical School, University of Bristol, Bristol, BS8 2BN, UK

**Keywords:** ALSPAC, Children of the 90s, birth cohort study, COVID-19, coronavirus, online questionnaire, antibody testing

## Abstract

The Avon Longitudinal Study of Parents and Children (ALSPAC) is a prospective population-based cohort study which recruited pregnant women in 1990-1992 and has followed these women, their partners (Generation 0; G0) and offspring (Generation 1; G1) ever since. The study reacted rapidly to the COVID-19 pandemic, deploying online questionnaires in March and May 2020. Home-based antibody tests and a further questionnaire were sent to 5220 participants during a two-week period of October 2020.

4.2% (n=201) of participants reported a positive antibody test (3.2% G0s [n=81]; 5.6% G1s [n=120]). 43 reported an invalid test, 7 did not complete and 3 did not report their result. Participants uploaded a photo of their test to enable validation: all positive tests, those where the participant could not interpret the result and a 5% random sample were manually checked against photos. We report 92% agreement (kappa=0.853). Positive tests were compared to additional COVID-19 status information: 58 (1.2%) participants reported a previous positive test, 73 (1.5%) reported that COVID-19 was suspected by a doctor, but not tested and 980 (20.4%) believed they had COVID-19 due to their own suspicions.  Of those reporting a positive result on our antibody test, 55 reported that they did not think they had had COVID-19.

Results from antibody testing and questionnaire data will be complemented by health record linkage and results of other biological testing– uniting Pillar testing data with home testing and self-report. Data have been released as an update to the original datasets released in July 2020. It comprises: 1) a standard dataset containing *all* participant responses to all three questionnaires with key sociodemographic factors and 2) as individual participant-specific release files enabling bespoke research across all areas supported by the study. This data note describes the antibody testing, associated questionnaire and the data obtained from it.

## Introduction

At the time of writing we are ten months into the coronavirus disease 2019 (COVID-19) pandemic and many countries have resorted to a second or third national lockdown, in an attempt to control the spread of the virus ^1^. It was noted early in the pandemic that antibody testing could give an indication of likely past exposure to the severe acute respiratory syndrome coronavirus 2 (SARS-CoV-2) infection ^2^. Recent studies on the prevalence of infection from antibody tests are primarily from hospital patients; yet mass testing in general populations is vital for improving the understanding of the spread of infection given that many individuals, particularly younger members of society, appear to be asymptomatic ^3^. The largest antibody testing study to date in England - the Real-time Assessment of Community Transmission (REACT), using 100,000 home-based antibody tests performed in June and July 2020 - suggests that 6% of the population had been infected with the virus ^4^. Testing in longitudinal population studies would be beneficial, in order to objectively identify cases and improve the assessment of the impact of, and risk factors for, infection on individuals who have rich pre-pandemic data and planned follow up. Work is ongoing in REACT to better understand the limitations of antibody testing, given the relative unknowns of when antibodies may begin to decline after infection, particularly in those with mild illness ^3^.

The Avon Longitudinal Study of Parents and Children (ALSPAC) is a unique multi-generational study: ‘G0’ includes the original pregnant women and their partners (mean age ~59 years); ‘G1’: the original index children (mean age ~28 years); and ‘G2’: the offspring of the original children ^5– 8^. ALSPAC has been able to collect self-reported data throughout the pandemic. This can be combined with data from clinical services based on linkage to medical and other records. In addition to collecting data about self-reported exposure to the infection and reporting on the impact of mitigation on participants (e.g. 9), we wanted to objectively estimate how many people in the study may have been infected with the virus that causes COVID-19. Antibody tests were therefore deployed to our participants.

This data note describes the data collected via our third online questionnaire in October 2020 which was complemented by home-based antibody testing. The update to the original dataset obtained from our first two online questionnaires ^10, 11^ are described here, together with any variables that have been derived using all sets of questionnaire data. We also present a summary of the antibody testing results and summarise the agreement between participant reports of their test results against our own checking.

## Methods

### Setting

ALSPAC is an intergenerational longitudinal cohort that recruited pregnant women residing in Avon, UK with expected dates of delivery 1 ^st^ April 1991 to 31 ^st^ December 1992 ^5, 6^. The initial cohort consisted of 14,541 pregnancies resulting in 14,062 live births and 13,988 children who were alive at 1 year of age. From the age of seven onwards, the initial sample was bolstered with eligible cases who had originally failed to join the study and there were subsequently 14,901 children alive at 1 year of age following this further recruitment ^7^. Please note, the study website contains details of all the data that is available through a fully searchable data dictionary and variable search tool.

ASLPAC developed a data collection strategy in response to the pandemic which was practical and yielded data rapidly. We achieved this through online only data collection approaches. This meant we had to restrict invites to those participants with a valid email address. This was coordinated alongside a systematic communications/outreach campaign to obtain updated information from our participants. Our questionnaires were developed and deployed using REDCap (Research Electronic Data CAPture tools ^12^); a secure web application for building and managing online data collection exercises, hosted at the University of Bristol.

### Invitation and reminder strategy for antibody testing

**Figure 1.**
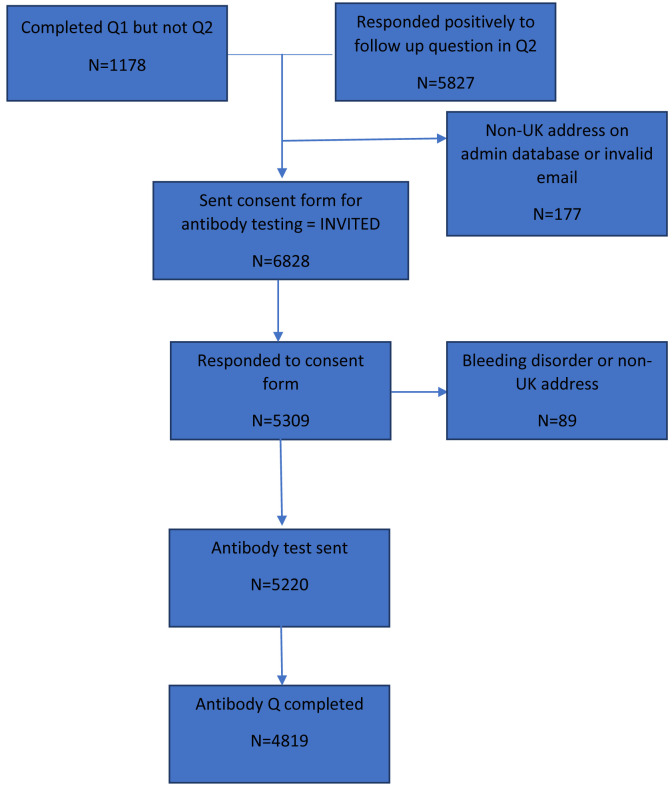
Flowchart showing number of participants invited to take part to those who took part. Q=questionnaire; UK=United Kingdom.

As part of the second questionnaire we asked participants if they were happy to be contacted about future research projects involving testing or taking biological samples. Participants who responded positively to this question (n=5,828, 90% of those responding to the questionnaire) and those who completed the first questionnaire but not the second (n=1,178), and therefore did not complete this question, formed the basis of our invites to take part in antibody testing ( Figure 1). An initial email was sent out to participants asking them to read a participant information sheet (PIS) and instruction booklet (which included a link to a brief video), containing details on the purpose of the research, what was involved and the risks of taking part. This information was based on a modified version of that used by the REACT study ^13^. Participants were asked to log on and complete an online REDCap consent form, which confirmed that they had read and understood the information provided, had the opportunity to ask questions and that they agreed to take part in the study. They also provided an address for the kit to be sent to. This may have differed to that stored on our administrative database and was kept only for the purpose of sending the test. Finally, participants were asked a screening question about bleeding disorders. Antibody tests were not sent to those participants who reported a bleeding disorder nor to those who provided an address outside the UK; costs and timescales made sending kits overseas impractical, resulting in 6,828 participants eligible to take part.

Between the 1 ^st^ and 5 ^th^ October 2020, 5,220 participants were sent testing kits through the post. An accompanying letter included detailed instructions and an invitation to complete an online questionnaire and upload a photo of the test result. Reminders were sent on the 9 ^th^ October. The questionnaire survey was live on the online platform for just over two weeks (all questionnaires and tests were completed between the 3 ^rd^ and 20 ^th^ October 2020). Unlike our standard questionnaires (usually completed annually) we did not provide any incentive for completion; however, we did offer a prize draw (three prizes of £100) for those who completed their questionnaire by 14 ^th^ October.

### Antibody tests

Una Health and Fortress Diagnostics Ltd. (Stoke-on-Trent, UK) supported this study by providing the antibody test cassettes procured initially by the Department of Health and Social Care, UK ^14^. The lateral flow test (Fortress Diagnostics, Antrim, Northern Ireland) was selected following evaluation of performance characteristics (sensitivity and specificity) against pre-defined criteria for detection of IgG ^15^, and extensive public involvement and user testing ^16^. Approval for the use of these kits for research purposes was obtained from The Medicines and Healthcare products Regulatory Agency (MHRA).

The lateral flow test (LFT) kits require the user to place a drop of blood onto the test, adding some buffer solution and waiting for around 15 minutes. The test could either be negative, positive (showing antibodies; IgG and/or IgM) or invalid, meaning that the test didn’t work. IgM antibodies appear first in infected individuals and levels typically fall rapidly, indicating recent infection. Whereas IgG may remain detectable for many months. We therefore considered a test positive if it showed the presence of IgG antibodies (with or without IgM).

### Questionnaire content

The questionnaire was considerably shorter than our previous COVID-19 questionnaires and captured information on the following:

●   Symptoms of COVID-19 and negative control symptoms since March 2020 (symptoms repeated from Q2)

●   Diagnosis with COVID-19 and testing history

●   Attempted and completed antibody test, with reasons why not attempted or completed

●   Result of antibody test and confidence in own interpretation

The final questionnaire (REDCap PDF) used is available with the associated data dictionary (which includes frequencies of all variables that are available) and both are available as extended data ^17^. In addition, participants were asked to take a photo of their test result and upload it through the online system if they wanted to (97% of responders did so).

### Validation of test results

Following the REACT protocol ^16^, two authors (KN and RH) reviewed a sample of photographs from all participants who reported a positive (IgG and IgG/IgM) result or stated they couldn’t tell, alongside a 5% random sample of the remainder. The two authors examined a random set of photos and recorded their interpretation of the results. These results were then compared to the participants’ reports. Agreement between authors and participants were assessed using kappa statistics.

## Key results

### Response rate

A total of 6,828 consent forms were sent to participants asking if they would be interested in completing a serology test and related questionnaire ( Figure 1). This group is considered the invited group. Of these, 5,220 serology kits and questionnaires were sent out (76% of those invited), of which 4,819 participants returned a questionnaire (71% of those invited; 92% of those sent a test kit and questionnaire; see Figure 1 for a flow diagram of participant numbers).

Female participants completed a larger proportion of questionnaires (73%). However, there was only a small difference in response rates for the sexes, with 67% of males and 72% of females who were invited returning the questionnaire. Table 1 summarises the response rate within each group organised by cohort structure. Of those invited to take part, 52% were G0s and 48% were G1s. It should be noted that considering those who were sent an antibody kit as the baseline (as opposed to those who were sent a consent form), the response rate was 92%.

**Table 1.** Number of participants who were invited and who responded to the third coronavirus disease 2019 (COVID-19) questionnaire.

Cohort Group	Sent a consent form - invited	Antibody test and questionnaire sent	Completed questionnaire ^1^
G0 Mothers	2637	2058	1944 (74%)
G0 Fathers/partners	995	747	709 (71%)
G1 Offspring daughters	2198	1706	1528 (70%)
G1 Offspring sons	883	618	554 (63%)
G1 Offspring partners (female)	61	50	46 (70%)
G1 Offspring partners (male)	54	41	38 (70%)
**TOTAL**	**6828**	**5220**	**4819 (71%)**

^1^ Proportions of those invited (i.e. those eligible)

Characteristics of responders according to key variables that will be released with the complete dataset can be seen in Table 2. The population who responded were predominantly white (> 98%) and the majority had at least A-level qualifications (optional exams sat at the age of 18 years), with almost 80% of the G1 cohort in this category. G0 Fathers/partners were three years older on average than G0 mothers (61.1 years vs 58.5 years) and G1 partners were two years older than G1 participants on average (30.3 years versus 28.2 years).

**Table 2.** Summary of key characteristics for those who responded. n (%) for categorical variables or mean (standard deviation; SD) for continuous variables.

	G0 Mothers	Go Fathers/ partners	G1 Offspring	G1 Offspring partners
Age (years)	58.5 (4.4)	61.1 (4.8)	28.2 (0.6)	30.3 (4.3)
Latest BMI ^1^	26.3 (4.9)	27.4 (4)	24.8 (5.3)	30 (4.7)
Latest Systolic BP ^1^	119.5 (14.2)	132.9 (13.1)	115.2 (10.9)	114.9 (12.2)
Latest Diastolic BP ^1^	70.5 (9.4)	77.4 (8.8)	67 (7.9)	65.4 (10.4)
Education level ^2^ ≥A level	1043 (56.1%)	472 (70.7%)	1328 (79.9%)	22 (64.7%)
Ethnicity ^3^ White	1826 (98.5%)	665 (99.6%)	1803 (97%)	Not available

^1^Data taken from the most recent clinic that individual attended where available

^2^Data taken from pregnancy questionnaires for G0 and from most recent questionnaire for G1 where available

^3^Data taken from pregnancy questionnaires for all

**Figure 2.**
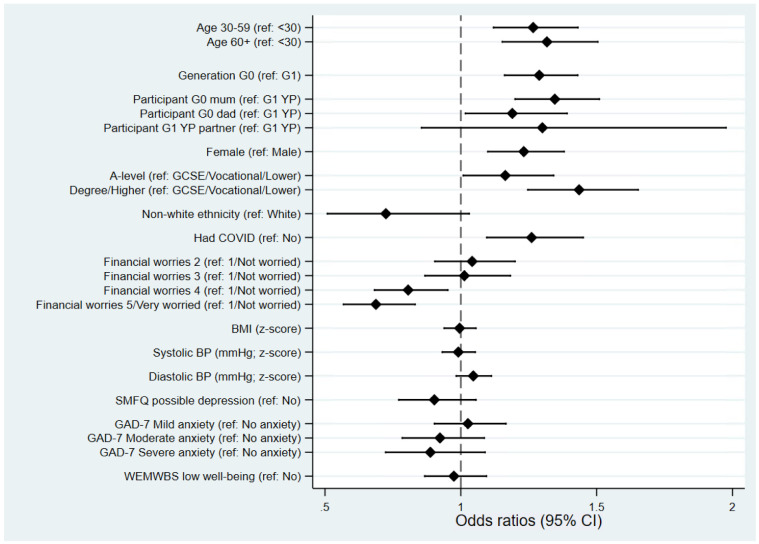
Forest plot describing the factors predicting completing the third coronavirus disease 2019 (COVID-19) questionnaire, of those invited. All results are odds ratios from logistic regression models with ‘completing questionnaire 3’ as the outcome, based on all participants who were invited ( *n* = 6,828; completed *n* = 4,819). Other than ‘age’, ‘generation’ and ‘participant’ (which are univariable models), all models are adjusted for ‘generation’ (G0 vs G1). Results to the right of the dashed line indicate increased odds of completing questionnaire 3 relative to the reference category, while results to the left indicate decreased odds. BMI=body mass index; BP=blood pressure; SMFQ=Short Mood and Feelings Questionnaire; GAD-7=Generalised Anxiety Disorder Assessment; WEMWBS=Warwick-Edinburgh Mental Wellbeing Scale; CI=confidence interval.

As with the previous questionnaire we sought to assess potential reasons for non-completion of this third COVID-19 questionnaire, which could potentially bias comparisons between questionnaire waves. Associations between various sociodemographic factors and returning this questionnaire, of those invited, were examined ( Figure 2). Returning the third questionnaire was strongly associated with age and generation whereby older, and therefore G0 participants, were more likely to complete compared to the younger/G1 participants. After adjusting for generation (G0 vs G1), there was evidence of social bias in those returning the questionnaire. Participants with higher education qualifications (a proxy for socioeconomic position) were much more likely to return the third questionnaire, while greater financial worry was associated with non-completion. However, physical and mental health were not strongly associated with the return of this questionnaire. As with the previous COVID-19 questionnaires ^10, 11^, women were more likely to return the questionnaire than men. Individuals who had previously self-reported that they had had COVID-19 (from either a positive test, doctor suspicions or own suspicions) were more likely to complete this third questionnaire. Participants from a non-white ethnic background were less likely to complete this questionnaire, although the 95% confidence intervals slightly overlap with the null.

### Test validation

Table 3 shows the agreement between each author’s and the participant’s interpretations for each category of result. Overall, there was 92% agreement between authors and participants (kappa=0.853). This can be broken down as follows: 99% agreement for the negative test results and 94% agreement for the IgG positive test results. The biggest disagreement was in the ‘can’t tell’ category, where the authors interpreted the result in all but two cases (removing those results in a kappa of 0.923). Of those participants who reported a positive IgG result but where the author disagreed, nine were negative, two were IgM positive and in one case the authors could not interpret the result. Of those participants who reported they could not tell but where the author disagreed, 17 were negative, one was IgM positive and three were IgG positive. We have created a new variable that replaces the participant’s report of the result with our own interpretation. Ten participants did not answer this question but did upload an image; these images were assessed, and the results have been added to the ‘ALSPAC interpretation of test results’ variable.

**Table 3.** Agreement between author and participant interpretation of antibody test results.

Test result	Author 1/participant (% agreement)	Author 2/participant (% agreement)	Total/participant (% agreement)
Negative	111/112 (99%)	120/122 (98%)	231/234 (99%)
IgM +ve	1/1 (100%)	0/0 (100%)	1/1 (100%)
IgG +ve ^a^	100/105 (95%)	87/94 (93%)	187/199 (94%)
Invalid	0/0 (100%)	1/1 (100%)	1/1 (100%)
Can’t tell	0/10 (0%)	2/13 (15%)	2/23 (9%)
**Total**	213/228(93%) kappa=0.869	210/230 (91%) kappa= 0.836	423/458 (92%) kappa=0.853
**Total removing ** **can’t tell**	213/218 (98%) kappa=0.946	208/217 (96%) kappa=0.917	421/435 (97%) Kappa= 0.923

^a^Two possible options were available -with or without IgM +ve, these have been combined

### Test results

Table 4a and Table 4b present the test results reported by participants ( Table 4a) and then the ALSPAC-validated results ( Table 4b). For the latter, 168 participants had a positive IgG result and 33 had a positive IgM and IgG result, meaning that 4.2% of participants reported a positive antibody test. 44 participants had an invalid result, one participant did not take a photo and could not remember their result when they came to complete the questionnaire, and even after validation by authors there were 10 cases where it was impossible to tell the result. Seven participants did not complete the test and three did not report on the test result. In total, 3.2% of G0s reported an IgG positive antibody test compared to 5.6% of G1s. Table 5 reports on the breakdown of key variables according to whether participants reported a positive test or not (with a positive result defined as either an ‘IgG positive’ or ‘IgG and IgM positive’ result, while a negative result is defined as either a ‘negative’ or ‘IgM positive’ result). No substantial differences were observed, although among the G0 generation participants with a positive result were slightly younger than participants with a negative result.

**Table 4a.** Antibody test result, split by generation (ALSPAC-interpreted).

	G0 – parents (%)	G1 – offspring (+partners; %)	Total (%)
Negative	2,501 (94.5%)	1,995 (92.2%)	4,496 (93.5%)
IgM positive	36 (1.4%)	21 (1%)	57 (1.2%)
IgG positive	67 (2.5%)	101 (4.7%)	168 (3.5%)
IgG and IgM positive	14 (0.5%)	19 (0.9%)	33 (0.7%)
Invalid	21 (0.8%)	23 (1.1%)	44 (0.9%)
Can’t tell result	7 (0.3%)	3 (0.1%)	10 (0.2%)
Not sure, as photo not taken	0 (0%)	1 (0.05%)	1 (0.02%)
Total	2,646	2,163	4,809

**Table 4b.** Antibody test result, split by generation (raw participant responses).

	G0 – parents (%)	G1 – offspring (+partners; %)	Total (%)
Negative	2,480 (94.1%)	1,985 (91.8%)	4,465 (93%)
IgM positive	35 (1.3%)	19 (0.9%)	54 (1.1%)
IgG positive	67 (2.5%)	100 (4.6%)	167 (3.5%)
IgG and IgM positive	16 (0.6%)	24 (1.1%)	40 (0.8%)
Invalid	20 (0.8%)	23 (1.1%)	43 (0.9%)
Can’t tell result	18 (0.7%)	11 (0.5%)	29 (0.6%)
Not sure, as photo not taken	0 (0%)	1 (0.05%)	1 (0.02%)
Total	2,636	2,163	4,799

**Table 5.** Antibody test result according to key variables, stratified by cohort. Differences assessed using chi-squared test for categorical variables and t-test for continuous variables; n (%) for categorical variables or mean (standard deviation; SD) for continuous variables. A positive result is defined as either an ‘IgG positive’ or ‘IgG and IgM positive’ result, while a negative result is defined as either a ‘negative’ or ‘IgM positive’ result.

	Positive result	Negative result	p-value
**Age (years)** G0 G1	58.2 (4.6) 28.2 (0.6)	59.2 (4.7) 28.3 (1.1)	0.077 0.763
**Gender** G0 – Male Female G1 – Male Female	18 (2.6%) 63 (3.3%) 29 (5.0%) 91 (5.9%)	681 (97.4%) 1856 (96.7%) 552 (95.0%) 1464 (91.2%)	0.355 0.442
**Education** G0 – O levels or lower A levels or higher G1 – O levels or lower A levels or higher	36 (3.6%) 44 (2.9%) 17 (5.0%) 81 (6.1%)	961 (96.4%) 1454 (97.1%) 323 (95.0%) 1252 (93.9%)	0.35 0.451
**BMI** G0 G1	27.1 (6.1) 24.6 (5)	26.5 (4.7) 24.9 (5.3)	0.372 0.491
**Systolic BP** G0 G1	121.9 (15.5) 116.5 (12.6)	123.1 (15.1) 115.2 (10.8)	0.528 0.216
**Diastolic BP** G0 G1	70.9 (9.1) 67.3 (8.3)	72.4 (9.8) 67 (8)	0.197 0.668

BMI=body mass index; BP=blood pressure

### Self-report of COVID-19

As with previous questionnaires, participants were asked whether they thought they had had COVID-19, prior to taking this antibody test. Options were: ‘Yes, confirmed by a positive test’, ‘Yes, suspected by a doctor but not tested’, ‘Yes, my own suspicions’ or ‘No’. In this questionnaire 58 (1.2%) respondents reported that they had tested positive for COVID-19, 73 (1.5%) reported that COVID-19 had been suspected by a doctor but not tested and 980 (20.4%) believed they had had COVID-19 due to their own suspicions. Table 6 summarises the responses to this question by cohort structure. Of those who reported a positive IgG result on the antibody test, 55 reported that they did not think they had had COVID-19. Further investigation is warranted to investigate symptom reports in this group to assess the proportion who are truly asymptomatic.

**Table 6.** Participant response as to whether they have had coronavirus disease 2019 (COVID-19).

	G0 – parents (%)	G1 – offspring (+partners; %)	Total (%)
Yes, positive test	17 (0.6%)	41 (1.9%)	58 (1.2%)
Yes, doctor suspected, no test	32 (1.2%)	41 (1.9%)	73 (1.5%)
Yes, own suspicions	472 (17.8%)	508 (23.5%)	980 (20.4%)
No	2128 (80.3%)	1573 (72.7%)	3701 (76.9%)
Total	2649	2163	4812

## Strengths and limitations of the data

This data collection has a number of strengths. Primarily, the study has been able to respond rapidly to the pandemic and collect several waves of data already. Secondly, the addition of antibody testing was unique in a longitudinal cohort study at the time of first publication. This puts us in an excellent position to identify true ‘cases’ of COVID-19 over and above self-report. We have undertaken linkage to Public Health England (PHE) Pillar I and II test results and are in the process of triangulating this data to obtain defined cases in our population. We achieved an excellent response rate despite a) the lack of incentives and b) the fact we were calling on our participants to take part in data collection for the third time in a matter of months. All the antibody tests were completed over a two-week period and therefore provide a relatively accurate snapshot in time We will use the data obtained to identify changes over time in symptom experience and better understand the waxing and waning of antibody levels according to likely date of infection. 

It should be noted, however, that a 2020 Cochrane review assessing the diagnostic accuracy of antibody tests identified a number of issues with the existing evidence ^18^, including that: 1) sensitivity of the tests is too low in the first week after symptom onset (this means we may have missed some cases who were in the early stage of infection in October), 2) the duration of the rise of antibodies is unknown due to the lack of data (current thinking is around 35 days after symptom onset; this means we may have missed cases who were infected early in the pandemic and may have since lost their antibodies) and 3) sensitivity of the tests has primarily been tested in hospital patients. The accuracy of home-based antibody tests in the general population is therefore still relatively unknown. We are not in a position to compare the results presented here with immuno-assays, so the population specific specificity-sensitivity is not possible to estimate. However, we have detailed survey data on symptoms, self-reported infection and also linkage to Pillar I and Pillar II testing as recorded by PHE for our G1 participants and those G0 mothers who have consented to such linkage. As noted above we will triangulate this data to identify true cases and when they may have occurred. Finally, all ALSPAC ‘cases’, together with both matched and random controls are currently being invited to take part in a sub-study as part of the UK Coronavirus Immunology Consortium (UK CIC, ^19^). This work will provide a better understanding of the immune response to the virus and help us with identifying true cases.

We were able to validate a sub-sample of test results using photographs of testing cassettes uploaded by the participants. We showed good agreement with participant reported results, which was comparable to that reported by REACT ^15^. In particular, we reported high agreement in those tests that were negative. However, we only took a 5% sub-sample of negative results and there is a possibility that in the remainder of the sample there were results reported as negative that were in fact positive. Based on the fact that two of the 5% sample we checked were in fact positive, there is the potential for approximately 40 tests in the whole sample to be incorrectly reported as negative. As we are not solely basing our case definition for future studies on the antibody test results there is every chance that we would pick such cases up through self-report of linkage to PHE test results, unless those participants were completely asymptomatic. Our agreement analysis suggests that in this population, participants were able to correctly determine their COVID-19 infection status most of the time.

We were able to assess some key sociodemographic factors predicting questionnaire completion, which is important for assessing and quantifying the extent of possible selection and collider bias, which may bias both our prevalence estimates and associations between variables ^20^. As reported both here and previously ^10, 11^, questionnaire response is socially patterned, with older, female, and higher-socioeconomic position participants more likely to respond. Additionally, in this questionnaire participants who previously reported that they had COVID-19 were more likely to respond. As those with COVID-19 were more likely to respond, our prevalence estimates may be somewhat inflated. Additionally, as previous COVID-19 status was associated with questionnaire completion (and therefore missing data), this could result in biases when assessing relationships between COVID-19 status and other risk factors which also predict questionnaire completion (e.g., age, sex, socioeconomic position). This study is therefore not generalisable to populations larger than ours that may contain the vulnerable groups most likely to benefit from self-screening programmes. Researchers using and interpreting this data should be aware of these potential biases ^21^.

The UK Scientific Advisory Group for Emergencies (SAGE) have noted potential behavioural responses to both positive and negative antibody results ^22^. This may have an impact on future research in this population. However, we were very careful to clearly explain to our participants that the test results were for research purposes and that they should not change their behaviour as a result. But this is not something we can guarantee.

## Data availability

### Underlying data

ALSPAC data access is through a system of managed open access. The steps below highlight how to apply for access to the data included in this data note and all other ALSPAC data:

1. Please read the ALSPAC access policy ^23^ which describes the process of accessing the data and samples in detail, and outlines the costs associated with doing so.

2. You may also find it useful to browse our fully searchable research proposals database ^24^, which lists all research projects that have been approved since April 2011.

3. Please submit your research proposal ^25^ for consideration by the ALSPAC Executive Committee. You will receive a response within 10 working days to advise you whether your proposal has been approved.

Please note that a standard COVID-19 dataset will be made available at no charge (see description below); however, costs for required paperwork and any bespoke datasets required additional variables will apply.

COVID-19 Questionnaire 3 Data File

Data from the third ALSPAC COVID-19 questionnaire (known internally as the serology questionnaire) is available in two ways.

1. A freely available standard set of data containing *all* participants together with key sociodemographic variables (where available) is available on request (see above). This dataset also includes data obtained from the first two COVID-19 questionnaires. Subject to the relevant paperwork being completed (costs may apply to cover administration) this dataset will be made freely available to any bona fide researcher requesting it. Variable names will follow the format *covid3_xxxx* where *xxxx* is a four-digit number. A full list of variables released is available here: https://doi.org/10.17605/OSF.IO/6JR7E. Frequencies of variables and details of any coding/editing decisions and derived variables are also available in the data dictionary: https://doi.org/10.17605/OSF.IO/6JR7E.
2. Formal release files have been created for G0 mothers, G0 fathers and G1 participants in the usual way and now form part of the ALSPAC resource (due to the small number of G1 partners contributing we will not be formally releasing this data, however, it may be available on request for specific G2 projects). These datasets (or sections therein) can be requested in the usual way. Variable names will replicate those in 1) above but as each variable in ALSPAC is uniquely defined we have added markers to denote the source of the variable. For example, in the above dataset, the age of the participant at completion (in years) is denoted by *covid3_9650*. In the mother’s dataset this will be denoted by *covid3m_9650*, for fathers/partner this will be *covid3p_9650* and for the G1 generation it will be *covid3yp_9650*. Frequencies for all variables for each participant group are available in the data dictionary in the usual way ^24^.

Text data and other potentially disclosive information will not be released until they have been coded appropriately. Data will be incorporated back into both file sets as they become available.

### Extended data

Open Science Framework: ALSPAC COVID-19 Questionnaires. https://doi.org/10.17605/OSF.IO/6JR7E ^17^

This project contains the following extended data:

1. Serology questionnaire: Covid_Antibody_Questionnaire.pdf (The final questionnaire; REDCap PDF)
2. Serology questionnaire: VariableList_COVID3.pdf (List of variable names and labels)
3. Associated data dictionary including frequencies of all variables that are available.

Data are available under the terms of the Creative Commons Attribution 4.0 International license (CC-BY 4.0).

## Consent

Participants consented electronically to take part in the antibody testing. Ethical approval for the study was obtained from the ALSPAC Ethics and Law Committee and the Local Research Ethics Committees. The South Central – Berkshire Research Ethics Committee provided specific approval for this data collection (REC reference number: 20/SC/0361). Informed consent for the use of data collected via questionnaires and clinics was obtained from participants following the recommendations of the ALSPAC Ethics and Law Committee at the time. Study participants have the right to withdraw their consent for elements of the study or from the study entirely at any time. Full details of the ALSPAC consent procedures are available on the study website ^25^.
