# Supplementary material for: The Avon Longitudinal Study of Parents and Children - A resource for COVID-19 research: Home-based antibody testing results, October 2020. An emphasis on self-screening at a population level
Source: Wellcome Open Res. 2021 Dec 2;6:34. Originally published 2021 Feb 18. [Version 2] doi: 10.12688/wellcomeopenres.16616.2 (PMC8453314; doi:10.12688/wellcomeopenres.16616.2)
Supplement: Peer review history

# Peer review: Reviewer response for version 1

**DOI:** 10.21956/wellcomeopenres.18318.r45653

**Published:** 2021-09-20

**Authors:** Citra N.Z. Mattar

**Affiliations:** 1 Yong Loo Lin School of Medicine, National University of Singapore, Singapore, Singapore

Thank you for the opportunity to review this paper by Northstone et al. The authors describe a process by which they performed a survey/questionnaire-based observational study on a sub-cohort of patients who were asked to administer a home test kit and report their observed results, as a trial run of self-screening for current COVID-19 infection.

**Positive aspects:**

- The authors have rapidly organized this sub-study to take advantage of their large ALSPAC cohort, and have screened a large number of participants with the antibody test. The response and participation rate is high, and attrition or exclusion rate is low.
- The questionnaire accompanying the self-administered screen allows the authors to contextualize the results and determine the likelihood of current and past COVID-19 infections.
- Test results were validated by photographs in a 5% random sample and demonstrated a high agreement between reviewer and participant, indicating fairly good test reliability.
- This is a good example of how certain populations can be screened rapidly and reliably for COVID-19 via electronic surveys and self-read screening tests, and appears to be efficiently replicable in populations of similar demographics.

**Negatives:**

 As the authors discussed, the study population is the main limitation, being almost entirely Caucasian and >50% completing at least A levels, with additional biases of higher socio-economic position, female gender and previous self-reported COVID-19 infections, demonstrating strong motivation to take the test and complete the study. Therefore this study is not generalisable to larger heterogenous populations which contain the vulnerable groups more likely to benefit from such self-screening programmes. The data generated and influence on health policy is thus potentially limited to similar populations.

 To me, this paper comes across as a report on the execution of an observational questionnaire-based study of a sub-cohort of the ALSPAC cohort, with secondary reporting of results. The data is useful for clinical screening, and generates epidemiological interest, and thus should be presented primarily as the value of self-reporting of self-administered home screening kits to screen for current or past infections, reliability of self-screening and reporting based on this cohort, biases, what this means for community screening. The data is interesting from the health policy perspective and primary care perspective so should be restructured to appeal to a wider clinical audience. This ought to be reflected in a more specific title emphasizing self-screening, and the abstract should clearly state the aims of determining the value of self-screening from the outset. The paper should perhaps explain in greater depth the value of this intervention and how it can be generalised or adapted to more diverse populations.

Are sufficient details of methods and materials provided to allow replication by others?

Yes

Is the rationale for creating the dataset(s) clearly described?

Yes

Are the datasets clearly presented in a useable and accessible format?

Yes

Are the protocols appropriate and is the work technically sound?

Yes

Reviewer Expertise:

Obstetrics, Maternal medicine, Fetal medicine, Gene Therapy, COVID in pregnancy

I confirm that I have read this submission and believe that I have an appropriate level of expertise to confirm that it is of an acceptable scientific standard, however I have significant reservations, as outlined above.

# Response

**Published:** 2021-11-26

**Authors:** Kate Northstone

**Affiliations:** University of Bristol, UK

We thank all reviewers for taking the time to read and comment on our data note describing the results from our first round of Covid-19 antibody testing in the ALSPAC cohort.

 We are delighted to see that reviewer 2 has approved the submission.

 In response to both reviewers 1 and 3, we would firstly like to emphasise that this is a data note rather than a research paper. Data notes have the primary aim of describing a dataset rather than presenting epidemiological research. Our responses therefore have this at the forefront.

 We provide reviewer 3’s comments below with our response in italics:

**We thank the reviewer for highlighting the positives of this study and will respond only to the negatives as follows.**

**Negatives:**

 "As the authors discussed, the study population is the main limitation, being almost entirely Caucasian and >50% completing at least A levels, with additional biases of higher socio-economic position, female gender and previous self-reported COVID-19 infections, demonstrating strong motivation to take the test and complete the study. Therefore this study is not generalisable to larger heterogenous populations which contain the vulnerable groups more likely to benefit from such self-screening programmes. The data generated and influence on health policy is thus potentially limited to similar populations."

*We completely agree and have now made this clear in the ‘strengths and limitations’ section (penultimate paragraph)*.

 "To me, this paper comes across as a report on the execution of an observational questionnaire-based study of a sub-cohort of the ALSPAC cohort, with secondary reporting of results. The data is useful for clinical screening, and generates epidemiological interest, and thus should be presented primarily as the value of self-reporting of self-administered home screening kits to screen for current or past infections, reliability of self-screening and reporting based on this cohort, biases, what this means for community screening. The data is interesting from the health policy perspective and primary care perspective so should be restructured to appeal to a wider clinical audience. This ought to be reflected in a more specific title emphasizing self-screening, and the abstract should clearly state the aims of determining the value of self-screening from the outset. The paper should perhaps explain in greater depth the value of this intervention and how it can be generalised or adapted to more diverse populations."

*The reviewer is correct in that the aim of the data note is to describe the data we have collected. However, the aim of that data collection was not to assess self-screening ability.*

*We appreciate the helpful comments on restructuring to appeal to a wider audience and have made some minor changes as a result.  We have amended the title adding reference to self-screening and have also added some detail throughout the paper as suggested:introduction (final sentence) and discussion (end of third para) (We are unable to add to the abstract as we are at the word limit). However, given the widespread use of lateral flow tests in the home, in the UK at least, we feel that we are a little too late to add to the knowledge base here and prefer to maintain the focus on describing our data and how it, together with both previous and subsequent data collections can help us to identify likely COVID-19 cases in our population and understand the waxing and waning of antibodies according to date of infection.*

# Peer review: Reviewer response for version 1

**DOI:** 10.21956/wellcomeopenres.18318.r45655

**Published:** 2021-09-14

**Authors:** Jean-Luc Murk

**Affiliations:** 1 Medical Microbiology and Immunology, Elisabeth-Tweesteden Ziekenhuis, Tilburg, The Netherlands

Dr. Northstone *et al.* have submitted a manuscript that describes their COVID-19 research in a British population based-cohort, that is followed since 1990. The COVID-19 study consists of elaborate online questionnaires and the results from a home-based SARS-CoV-2 IgM and IgG test. The study was performed in 2020. The antibody test results give an indication of the percentage of SARS-CoV-2 infections up to period of testing (October 2020). The research is technically sound and the datasets contain a lot of interesting information.

Are sufficient details of methods and materials provided to allow replication by others?

Yes

Is the rationale for creating the dataset(s) clearly described?

Yes

Are the datasets clearly presented in a useable and accessible format?

Yes

Are the protocols appropriate and is the work technically sound?

Yes

Reviewer Expertise:

medical microbiology, serology, virology, COVID-19

I confirm that I have read this submission and believe that I have an appropriate level of expertise to confirm that it is of an acceptable scientific standard.

# Response

**Published:** 2021-09-16

**Authors:** Kate Northstone

**Affiliations:** University of Bristol, UK

Thank you very much for taking the time to read and comment on our data note. We are delighted to see that you have approved the submission.

# Peer review: Reviewer response for version 1

**DOI:** 10.21956/wellcomeopenres.18318.r45611

**Published:** 2021-09-07

**Authors:** Morteza Taheri

**Affiliations:** 1 Department of Sport Sciences, Imam Khomeini International University, Qazvin, Iran

**Abstract:**

- Statistical values ​​should be stated in the results section of the abstract. 
- Your abstract should be organized in such a way that the content of the aim, methodology, findings and conclusion are stated separately.
- Key words should be checked based on Mesh standard.
- The general conclusion in the abstract should be more clearly stated.

**Introduction:**

- In addition to the explanations related to the topic of Covid-19 epidemics and the need for diagnostic antibody tests, it is also worth mentioning their effectiveness in vaccination, which you can use from the following references: Dergaa *et al. *(2021 ^1^).
- The introduction is very brief, it is better to be more complete and focus more on international survey study with respect to Covid-19. Use the following reference for showing the importance of survey by using a large number of questionnaires: Trabelsi *et al.* (2021 ^2^).
- You mentioned less about the importance of addressing the issue of Covid-19 in the text, so it is important to use up-to-date references regarding it.

 

**Method:**

- The validity and reliability of questionnaires?
- Exclusion and inclusion criteria for participants?
- Please kindly upload the questionnaire for consideration. 
- Any ethical committee code?

**Result:**

- Use a graph to show the results more clearly.
- Specify the statistics method in tables. (e.g. t-test, etc.).

 

**Discussion:**

- Justify and discuss the results more clearly. 
- Report recommendations for future studies based on study limitation(s). 

Are sufficient details of methods and materials provided to allow replication by others?

Partly

Is the rationale for creating the dataset(s) clearly described?

Yes

Are the datasets clearly presented in a useable and accessible format?

Yes

Are the protocols appropriate and is the work technically sound?

Yes

Reviewer Expertise:

sport medicine and Covid-19.

I confirm that I have read this submission and believe that I have an appropriate level of expertise to confirm that it is of an acceptable scientific standard, however I have significant reservations, as outlined above.

## References

[1] : COVID-19 Vaccination, Herd Immunity and The Transition Toward Normalcy: Challenges with The Upcoming Sports Events. *Ann Appl Sport Sci.* .2021;9(3) : Reference source

[2] : Sleep Quality and Physical Activity as Predictors of Mental Wellbeing Variance in Older Adults during COVID-19 Lockdown: ECLB COVID-19 International Online Survey. *International Journal of Environmental Research and Public Health* .2021;18(8) : 10.3390/ijerph18084329 10.3390/ijerph18084329 PMC807384533921852

# Response

**Published:** 2021-11-26

**Authors:** Kate Northstone

**Affiliations:** University of Bristol, UK

We thank all reviewers for taking the time to read and comment on our data note describing the results from our first round of Covid-19 antibody testing in the ALSPAC cohort.

 We are delighted to see that reviewer 2 has approved the submission.

 In response to both reviewers 1 and 3, we would firstly like to emphasise that this is a data note rather than a research paper. Data notes have the primary aim of describing a dataset rather than presenting epidemiological research. Our responses therefore have this at the forefront.

 We provide reviewer 1 and 3’s comments below with our response in italics:

**Reviewer 1:**

**Abstract:**

- Statistical values ​​should be stated in the results section of the abstract. 

*As the focus of this article is a description of the data we do not feel this is appropriate.*

  

- Your abstract should be organized in such a way that the content of the aim, methodology, findings and conclusion are stated separately.

*We feel that the abstract is an appropriate summary of the data note, follows the author guidelines and is aligned to all the other data notes we have published relating to Covid-19 data collection.*


* *

- Key words should be checked based on Mesh standard.

*Thank you, we have done that and are ensuring consistency also with our previous and subsequent data notes.*

  

- The general conclusion in the abstract should be more clearly stated

*As a description of the data there is no firm conclusion.*

**Introduction:**

- In addition to the explanations related to the topic of Covid-19 epidemics and the need for diagnostic antibody tests, it is also worth mentioning their effectiveness in vaccination, which you can use from the following references: Dergaa *et al. *(2021 ^1^ ).

*At the time of writing and initiating the testing programme in our population, home-based tests were relatively new and our testing was carried out well before any vaccinations had been approved for use. As noted in our response to reviewer 3, the focus of the current study was to assist in establishing ‘caseness’ in our population (avoiding reliance on self-report when testing was not widespread) rather than building the case for the use of home testing in the community.*

  

- The introduction is very brief, it is better to be more complete and focus more on international survey study with respect to Covid-19. Use the following reference for showing the importance of survey by using a large number of questionnaires: Trabelsi *et al.* (2021 ^2^ ).

*Again, our focus is here is on describing the data we have. The current data note is one of several describing the detailed resource that ALSPAC now has and contributes one of five questionnaires that have now been completed by our participants. Given the time that has passed we would prefer to keep the data note as is so that our contribution flows in a succinct way* *.*

- You mentioned less about the importance of addressing the issue of Covid-19 in the text, so it is important to use up-to-date references regarding it.

*As above we are describing our dataset and our response to the pandemic..*

**Method:**

- The validity and reliability of questionnaires?

*As questionnaires had to be designed very quickly given the sudden onset of the pandemic we were not able to formally test the validity or reliability of our questionnaires. However, the questions used are very similar to many other longitudinal population studies in the UK, with whom we are now working closely. Indeed, the need to collect more objective data was driven by the subjective nature of participants ‘self-suspicions’ of COVID-19 infection at a time when testing was not widespread and these antibody tests can be used to test the validity and reliability of the questionnaires (see Table 6).*

  

- Exclusion and inclusion criteria for participants?

*This is described in the invitation strategy section:*

*Inclusion: “* *As part of the second questionnaire we asked participants if they were happy to be contacted about future research projects involving testing or taking biological samples. Participants who responded positively to this question (n=5,828, 90% of those responding to the questionnaire) and those who completed the first questionnaire but not the second (n=1,178), and therefore did not complete this question, formed the basis of our invites to take part in antibody testing.”*

*Exclusions: “* *Antibody tests were not sent to those participants who reported a bleeding disorder nor to those who provided an address outside the UK* *“*

*Please also see Figure 1.*

  

- Please kindly upload the questionnaire for consideration. 

*As described in the “Questionnaire content” section the Q is available in the uploaded data dictionary. Please see:* *https://osf.io/6jr7e/* *.*

  

-  Any ethical committee code?

*This is also provided in the consent section: “* *REC reference number: 20/SC/0361”.*

**Result:**

- Use a graph to show the results more clearly.

*We are not sure which results you are referring to. All the tables presented are simple and one graph is used which presents the more detailed information.*

  

- Specify the statistics method in tables. (e.g. t-test, etc.).

*The tables that present the results of statistical tests clearly state already in the table or in the title which test is used (Table 3 using kappa and Table 5 using chi squared or t-tests).*

**Discussion:**

- Justify and discuss the results more clearly. 

*We are not sure which results the reviewer is referring to specifically. In the discussion we have summarised the results and detailed key strengths and limitations as appropriate for a data note. If the reviewer has more specific comments we would be happy to update as appropriate.*

- Report recommendations for future studies based on study limitation(s). 

*As* *this data collection was time critical and is now a year old we are not sure that this is helpful, particularly as this is a data note not a research paper.*
